# Semi-automatic segmentation of myocardium at risk from contrast enhanced SSFP images - validation against manual delineation and SPECT

**DOI:** 10.1186/1532-429X-17-S1-Q127

**Published:** 2015-02-03

**Authors:** Jane Tufvesson, Marcus Carlsson, Anthony H Aletras, Henrik Engblom, Jean-Francois Deux, Sasha Koul, Peder Sörensson, John Pernow, Dan Atar, David Erlinge, Håkan Arheden, Einar Heiberg

**Affiliations:** 1 Cardiac MR group Lund, Dept. of Clinical Physiology, Lund University, Lund, Sweden; 2 Dept of Numerical Analysis, Faculty of Engineering, Lund University, Lund, Sweden; 3 Dept of Cardiology, Henri Mondor Hospital, Creteil, France; 4 Dept of Cardiology, Lund University, Lund, Sweden; 5 Dept of Medicine, Karolinska Institutet, Karolinska University Hospital, Stockholm, Sweden; 6 Dept of Cardiology B, Oslo, University Hospital Ullevål and Faculty of Medicine, University of Oslo, Oslo, Norway

## Background

The development of treatments to limit myocardial injury in patients with acute STEMI is dependent on methods that accurately determine the amount of mycoardium at risk (MaR). Both T2-weighted imaging and contrast enhanced SSFP (CE-SSFP) have been validated against SPECT and can determine the MaR by CMR one week after an infarct. CE-SSFP has recently been used in two multi-center studies^1,2^. An automatic algorithm for quantification of MaR from T2-weighted images has previously been described but not been tested in CE-SSFP. The aim of this study was to further develop and validate this automatic method for CE-SSFP.

## Methods

The automatic algorithm, called Segment MaR, defines the MaR region as the continuous region most probable of being MaR, by estimating the intensities of normal myocardium and MaR with an expectation maximization algorithm and restricting the MaR region by an a priori model of the maximal extent for the user defined culprit artery. The algorithm was modified to be applied at both end diastole and end systole.

The automatic algorithm was validated against manual delineation in 114 patients from two multi center studies (CHILL-MI [1] and MITOCARE [2]) and one single center study as well as SPECT in a sub population (n=16). Endocardial and epicardial borders as well as the hyperenhanced MaR region were manually delineated at end diastole and end systole.

MaR was quantified as percent of left ventricular mass (%LVM). Comparisons were done using Bland-Altman bias (mean ± standard deviation) and linear regression analysis (correlation coefficient).

## Results

**Figure 1:**
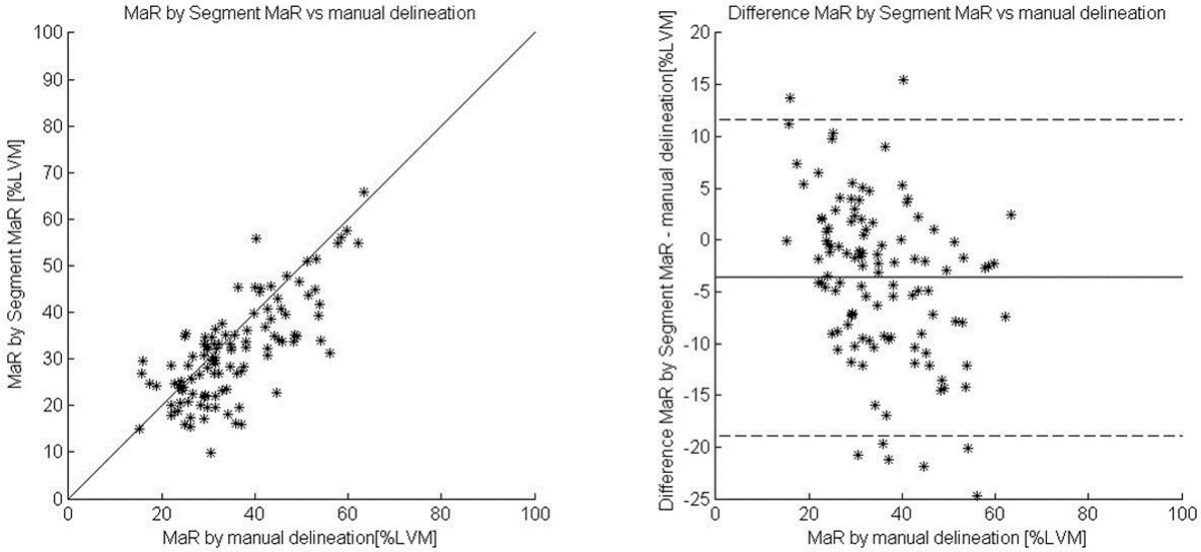
**Correlation and Bland-Altman plot for automatic segmentation against manual delineation in the test set.** Myocardium at risk (MaR) by automatic segmentation Segment MaR plotted against manual delineation as % of LVM (left) and difference between MaR by automatic segmentation Segment MaR and manual delineation (right).

**Figure 2:**
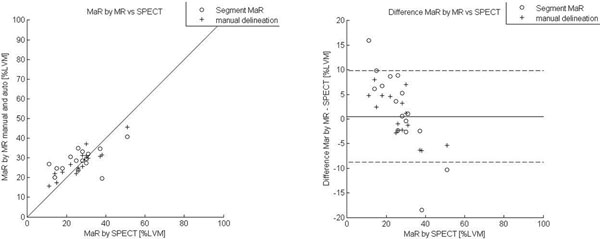
**Correlation and Bland-Altman plot for automatic segmentation and manual delineation against SPECT.** Myocardium at risk (MaR) by automatic segmentation Segment MaR and manual delineation in CE-SSFP against SPECT as % of LVM (left) and difference between MaR by CE-SSFP and SPECT (right).

MaR assessed by manual delineation was 35.2 ± 10.8 %LVM and MaR assessed by Segment MaR was 31.5 ± 10.6 % (n=114). Bias was -3.7 ± 7.7 % of LVM and the correlation was R=0.74 when Segment MaR was compared to manual delineation of MaR (Figure 1). In the smaller validation subset (n=16) a comparison between SPECT and CE-SSFP was performed for Segment MaR as well as manual delineation. There was a low bias, 0.5 ± 5.1 % of LVM and a correlation of R=0.88, between manual delineation in CE-SSFP and SPECT, and a bias of 1.9 ± 8.3 % of LVM and a correlation of R=0.56 between Segment MaR in CE-SSFP and SPECT (Figure 2).

## Conclusions

A good agreement was shown between automatic Segment MaR and manually assessed MaR in CE-SSFP CMR as well as compared to SPECT. The proposed algorithm seems to be a promising, objective method for standardized MaR quantification in CE-SSFP CMR.

## Funding

Swedish Research Council, Swedish Heart and Lung Foundation, The Medical Faculty of Lund University, Sweden, and Region of Scania, Sweden.
